# A novel device for elimination of cancer cells from blood specimens

**DOI:** 10.1038/s41598-020-67071-w

**Published:** 2020-06-23

**Authors:** Agnes Weth, Ilona Krol, Kurt Priesner, Cinzia Donato, Stefan Pirker, Christoph Wolf, Nicola Aceto, Werner Baumgartner

**Affiliations:** 1 Institute of Biomedical Mechatronics, Johannes Kepler University of Linz, Altenbergerstr. 69, 4040 Linz, Austria; 2 Cancer Metastasis Laboratory, Department of Biomedicine, University of Basel and University Hospital Basel, 4058 Basel, Switzerland; 3 Griesmühle Ltd, Griesmühlweg 14, 4111 Walding, Austria; 4 Department of Particulate Flow Modelling, Johannes Kepler University of Linz, Altenbergerstr. 69, 4040 Linz, Austria

**Keywords:** Cancer, Metastasis

## Abstract

Circulating tumor cells (CTCs) are derivatives of solid cancerous lesions that detach from the tumor mass and enter the blood circulation. CTCs are considered to be the precursors of metastasis in several cancer types. They are present in the blood of cancer patients as single cells or clusters, with the latter being associated with a higher metastatic potential. Methods to eliminate CTCs from the bloodstream are currently lacking. Here, we took advantage of the lower shear stress-resistance of cancer cells compared to blood cells, and developed a device that can eliminate cancer cells without blood damage. The device consists of an axial pump and a coupled rotating throttle, controllable to prevent local blood flow impairment, yet maintaining a constant shear performance. When processing cancer cells through our device, we observe cancer cell-cluster disruption and viability reduction of single cancer cells, without noticeable effects on human blood cells. When injecting cancer cell-containing samples into tumor-free recipient mice, processed samples fail to generate metastasis. Together, our data show that a selective disruption of cancer cells is possible while preserving blood cells, paving the way towards the development of novel, implantable tools for CTC disruption and metastasis prevention.

## INTRODUCTION

Despite remarkable advances in diagnosis, surgical techniques and anticancer therapies, cancer remains among the leading causes of death worldwide, with an estimated increase of 70% in the next 20 years according to the World Health Organization^1^. It is estimated that, annually, more than 8 million people die from cancer worldwide and over 90% of cancer-related deaths are due to metastasis development^2–4^, highlighting the unmet clinical need to develop new anti-metastatic therapeutic approaches. However, to date, the largest proportion of cancer research has been conducted with a focus on the primary tumor^5^ and as a consequence, our understanding of the vulnerabilities of metastatic cells remained limited, thus hampering the development of effective metastasis-suppressing agents. In contrast, a therapeutic approach hindering the development of metastasis may greatly improve the span and quality of life for cancer patients.

The main precursors of metastasis are considered to be circulating tumor cells (CTCs), i.e. cancer cells that detach from a solid cancerous lesion located anywhere in the body and enter the bloodstream^6,7^. Once in the blood circulation, CTCs navigate in the form of single cells, clusters of cells (CTC clusters) or clusters of CTCs and immune cells, able to reach distant sites and give rise to metastasis^7–9^. However, while several technologies are able to identify and isolate CTCs from human blood *ex situ*^10–13^ and *in situ*^14–16^, no methods are currently available to efficiently eliminate CTCs intravascularly, before they have reached the metastatic site. The before mentioned *in situ* methods are typically indwelling intravascular aphaeretic CTC isolation systems to continuously collect CTCs directly from a peripheral vein. The system returns the remaining blood products after CTC enrichment, permitting interrogation of relatively large blood volumes of up to 1–2% of the entire blood over 2 h. Approximately 70–90% of the CTCs are removed from this fraction of human blood^14^. While this is perfectly suited and highly valuable for diagnostic purposes to detect, quantify and characterise CTCs, this system is of limited use for therapeutic approaches aiming to effectively remove CTCs from the blood compartment.

Blood cells such as erythrocytes, leucocytes and thrombocytes are considered to be well acquainted to liquid shear stress due to their physiological role within the bloodstream. For instance, in arteries, typical physiological shear stress levels of 2–10 Pa can be reached^17,18^. CTCs, on the other hand, originate from solid tissues that do not experience high shear stress levels while located in the tumor. Recent studies have shown that CTCs carry different deformability properties and shear stress resistance compared to blood cells^19,20^, suggesting a potential window of opportunity for the development of CTC-disruption devices that do not affect the integrity of blood cells.

The cardiovascular system is highly controlled to ensure efficient local blood supply. Thus, introducing throttles, filters or pumps into the vascular system could affect blood flow and lead to undesirable local hypertension or hypotension. In contrast, we thought of constructing a non-pumping-pump, i.e. an axial pump coupled to a rotating throttle (referred to here as “CTC-disruption device”). Such a device is controllable in a way that allows operation without impairing the local blood flow, i.e. by adapting the flow rate while applying constant shear performance, which depends on the amplitude and the duration of the shear stress.

In this work, we combine engineering tools, cellular biology and mouse models to test the ability of a newly developed device to eliminate cancer cells from human blood samples. In the long run, our study aims at representing the first step towards the development of novel implantable devices for metastasis prevention.

## Results

**Figure 1:**
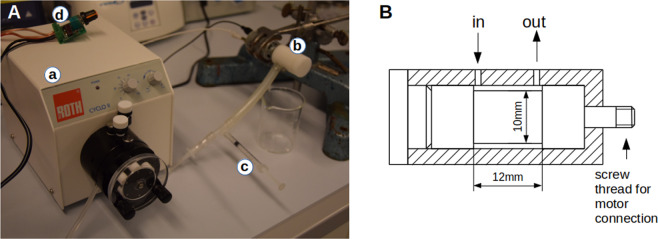
Setup to apply shear stress for different durations to suspended cells. The experimental setting is shown in (**A**). A roller-pump (a) pumps isotonic salt solution at a given rate into the rotating resistor (rotating throttle) which consists of a rotating cylinder in a concentric hole (b). Via a syringe (c) cells can be injected. The motor controller (d) allows the precise setting of the rotational speed and thereby of the shear stress while the pumping rate of pump (a) allows the setting of the duration of shear the cells obtain. The design of the rotating throttle is shown in (**B**).

We first sought to generate a novel device consisting of a pump and a rotating throttle, i.e. a rotating cylinder in a cylindric hole with a defined clearance. We considered two main parameters to be important for the impact of the shear onto cancer cells (serving as model system for CTCs) as well as onto blood cells: the duration and the magnitude of the applied shear stress. In order to find a useful window of opportunity, we started with a setup that consists of separated pump and throttle (Fig. 1A). Due to the separation of the pump and the throttle, the magnitude of shear stress and the duration can be set up virtually independently, as the axial shear stress can be neglected in comparison to the circular shear stress, induced by the rotation of the throttle (Fig. 1B).

**Figure 2:**
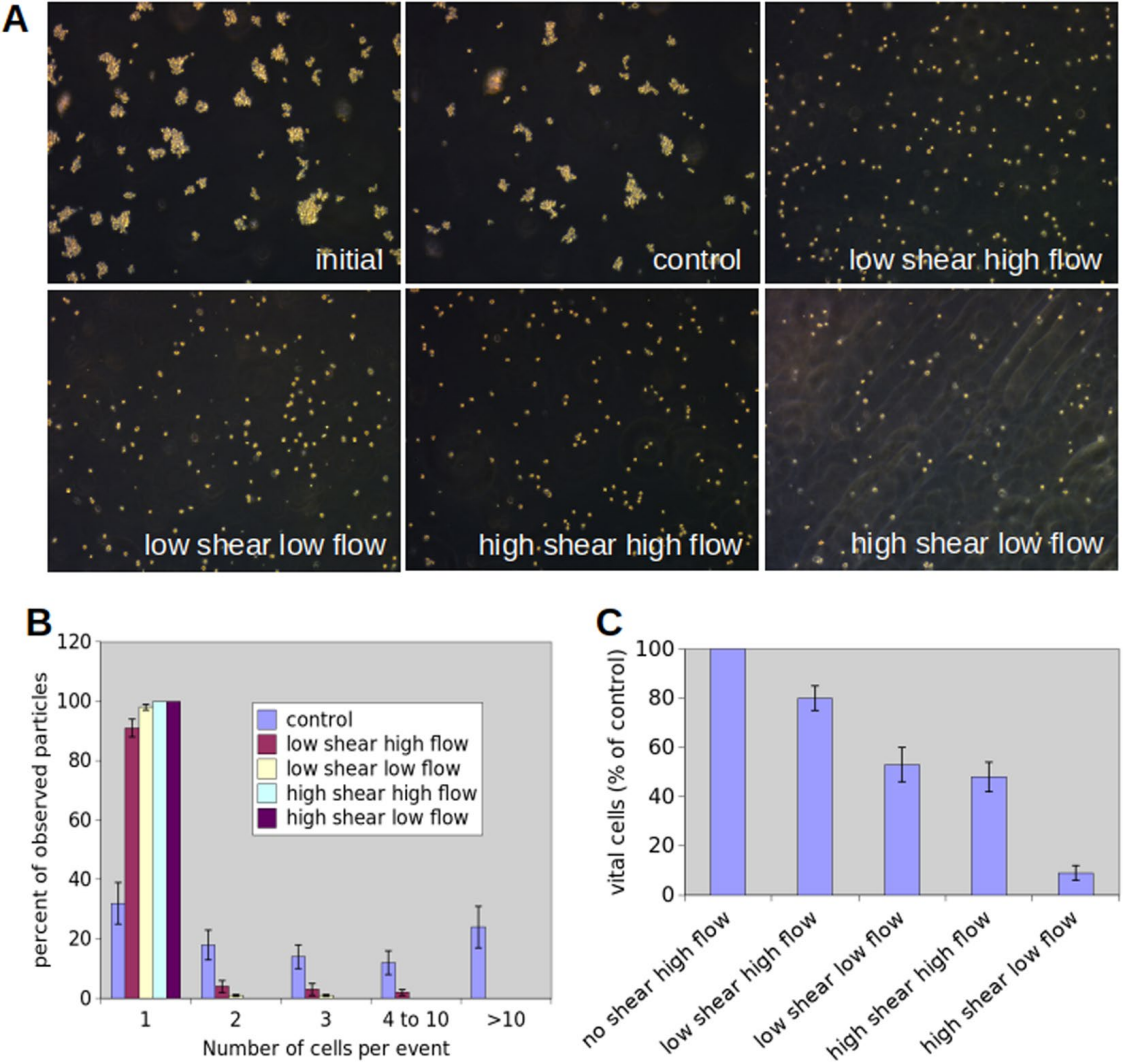
Characterization of cancer cells and cancer cell-clusters exposed to different shear stress conditions. (**A**) Darkfield images of cells and cell clusters exposed to different shear conditions. Cells were then exposed to either low shear stress of approximately 7,5 Pa or high shear stress of approximately 15 Pa. The time of exposure was adjusted by the flow rate of the pump. For the high flow rate the exposure time was approximately 0.12 s while for the low flow conditions the exposure time was approximately 0.24 s. **(B**) Distribution of cluster size. The number of cells per object seen in the dark field microscope (**A**) was counted. Under control conditions most objects are multicellular and only about 30% of the objects are single cells. Under mild shear exposition (low shear, high flow) more than 90% of the objects are single cells. Under stronger shear stress or longer exposure, virtually all objects are single cells. Using pairwise comparison with a χ^2^-test it can be found that low chear low flow and high shear high flow conditions differn not significantly while all other pairwise comparisons exhibit significant differences (p = 0.05). Besides the dissociation of clusters, single cancer cells become damaged as shown in (**C**). The viability of the cells was quantified photometrically. Control conditions were normalized to 100%. Under the highest shear exposure (high shear low flow) more than 90% of the CTCs are dead. These reductions in comparison to control (no shear) are highly significant when evaluated using a Wilcoxon-rank-sum-test at a significance level p < 0.01.

Using this setup, we tested whether our CTC-disruption device was able to destroy cancer cells while preserving the integrity of white blood cells (WBCs) and red blood cells (RBCs). To this end, based on recent studies^19,20^, we reasoned that shear stress resistance of cancer cells might be lower compared to blood cells, and aimed to find the lowest shear forces that would result in cancer cells disruption. Particularly, we tested different conditions for their ability to disrupt cancer cells cells in culture (Fig. 2). Untreated cells from tumor-derived cultures typically grow in clusters (see panel “initial”, Fig. 2A). For control measurements, cells were passed through the CTC-disruption device with the throttle not rotating, thus the cells were only exposed to the shear conditions of the pump and the piping. Cells were then either exposed to a low shear stress (7.5 Pa) or a high shear stress (15 Pa). The processing time was adjusted by adapting the flow rate of the pump. For the high flow rate, we used a processing time of 0.12 s, while for the low flow rate we used a processing time of 0.24 s. Upon processing, we observed a remarkable reduction in the number of cancer cell clusters in all tested conditions, highlighted by counting the number of cells within each cluster of different size (Fig. 2A,B). Particularly, we observed that in control conditions most objects are multicellular, with only about 30% of them being single cells. In contrast, under mild shear exposition (low shear, high flow) more than 90% of the objects were single cells, while under stronger shear stress or longer exposure virtually all objects were single cells (Fig. 2B). We then tested whether cell viability was altered upon passage through the CTC-disruption device. To this end, we measured viability with photometrical testing using neutral red uptake to the lysosomes, and normalizing all data to control conditions. We observe that the viability of the remaining single cancer cells decreased dramatically as a function of the applied shear power (shear stress and exposure time) (Fig. 2C). If the cancer cells were treated so that single cancer cell-suspensions were obtained, the drop of viability was even more pronounced. For high-shear-low-flow the viability dropped by 98%.

**Figure 3:**
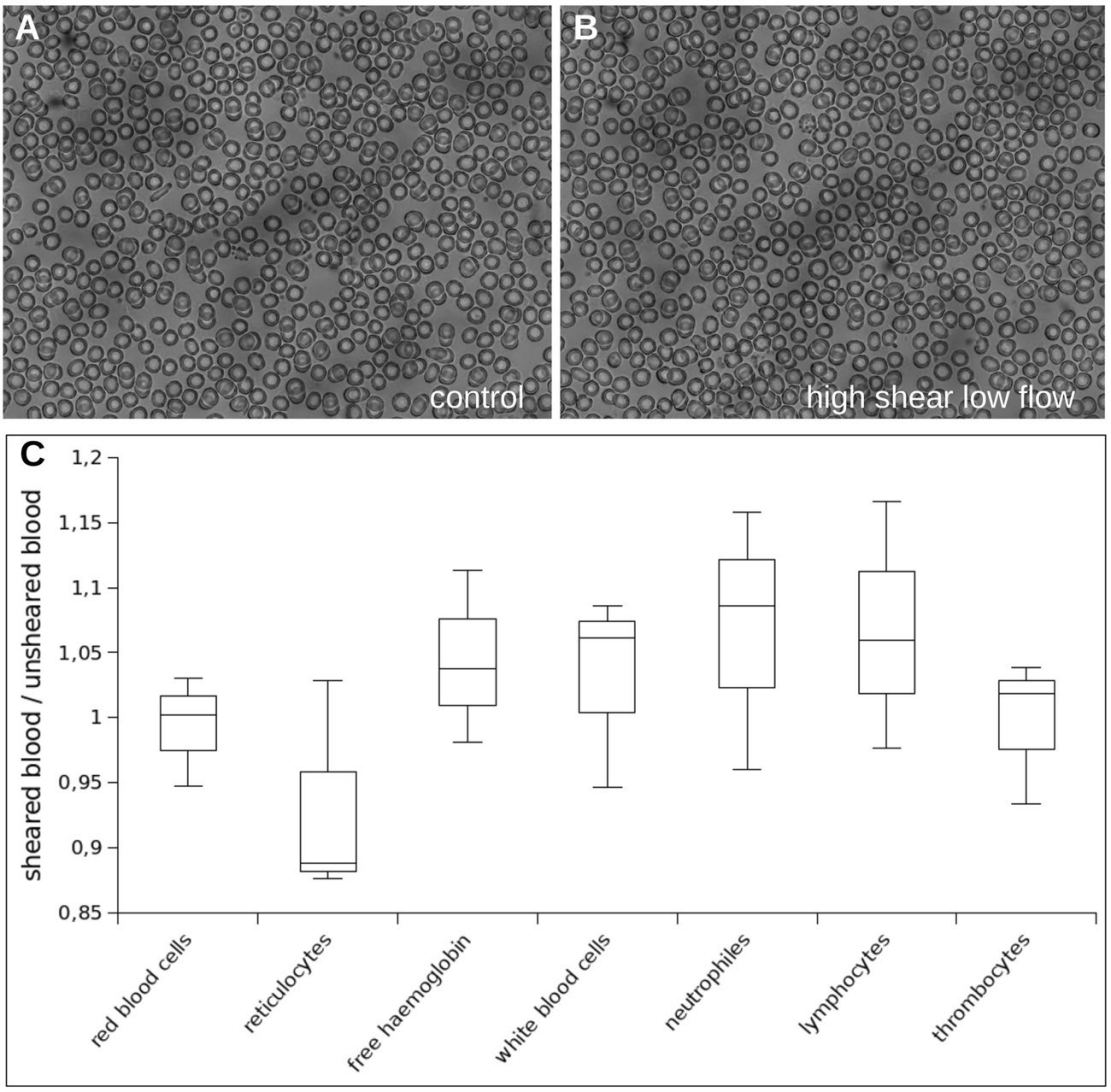
Characterization of blood exposed to shear stress. Blood of three healthy donors was used. Each sample was divided into 4 parts. One was used as untreated control while the others were exposed to different shear conditions. A blood smear of untreated blood is shown in (**A**). A smear of the blood of the same donor after passing the device at maximal shear stress is shown in (**B**). Untreated and sheared blood were analyzed using a hematology analyzer. The comparison of unsheared and sheared blood is shown in (**C**) where the ratio of the parameter-values of sheared and unsheared blood is plotted. All ratios are close to 1 and no significant differences were found (both using a one-sample t-test as well as a Wilcoxon-rank-sum-test at a significance level α = 0.05).

We then assessed the effects of the most severe conditions our device could deliver, i.e. 19 Pa shear stress for 0.24 s, on normal blood cells from healthy donor blood, run through our CTC-disruption device and then processed with a hematology analyzer and chemical analysis according to a standard clinical lab test. Strikingly, we found that treatment of blood cells with 19 Pa for 0.24 s did neither alter their composition nor their morphology (Fig. 3A–C). We also found no changes in free haemoglobin in the serum, concluding that we had no significant lysis of erythrocytes (Fig. 3C). Together, we conclude that shear stress magnitudes ranging from 7.5–15 Pa for up to 0.24 s are able to disrupt cancer cell clusters into single cancer cells, and that viability of the resulting single cancer cells drops down, while normal blood cells remain largely unaffected.

**Figure 4:**
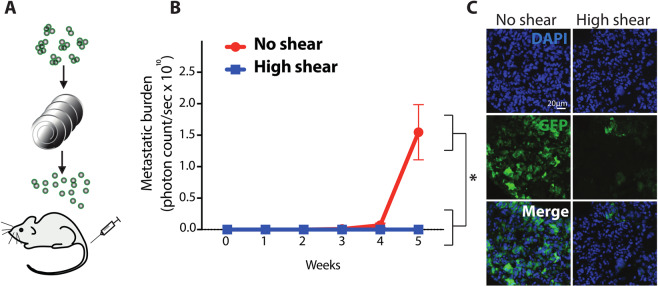
Characterization of metastatic potential of sheared versus unsheared cancer cells. Schematic of experimental design. Cancer cell clusters were exposed to shear stress, collected and injected in the tail vein of immunodeficient mice (**A**). The graph shows metastatic burden as a mean photon count/sec. n = 4 for no shear, n = 3 for high shear; error bars represent S.E.M., *P < 0.0019 by Student’s t test. (**B**). Representative images of lungs from mice injected with sheared versus unsheared CTCs and stained for GFP (green) and DAPI (blue) (**C**).

Upon demonstrating that CTCs are more susceptible to shear stress within our CTC-disruption device compared to normal blood cells, we sought to test whether cancer cells that went through the device were also less capable to seed a metastasis, compared to control cells. To assess this point, we focused on the specific condition of 15 Pa for 0.24 s, capable of killing 90% of cancer cells and disrupting all cancer cell clusters. We spiked a total of 1×10^7^ cancer cells labelled with GFP-luciferase (as a suspension of clusters) in PBS, processed them with our CTC-disruption device and then immediately injected the resulting cell suspension in the tail vein of recipient immunodeficient mice to measure metastasis development (Fig. 4A). Strikingly, during the course of a 5-week metastasis growth curve experiment, we observed a remarkable reduction in the total metastatic burden of mice that were injected with cancer cells that were processed with the CTC-disruption device, compared to control animals (Fig. 4B,C). To further validate this finding, we stained the lungs of all injected animals with antibodies against GFP (expressed only by the cancer cells) and consistently observed a high degree of metastatic foci in control mice, while no macro-metastases were detected in mice that were injected with cancer cells that underwent processing with our device (Fig. 4D). This does not only hold true for clusters, but also for single cancer cell-suspensions as shown in the supplementary data.

Together, our results demonstrate that cancer cells are more susceptible to high shear forces compared to blood cells, and that this differential susceptibility can be exploited therapeutically.

**Figure 5:**
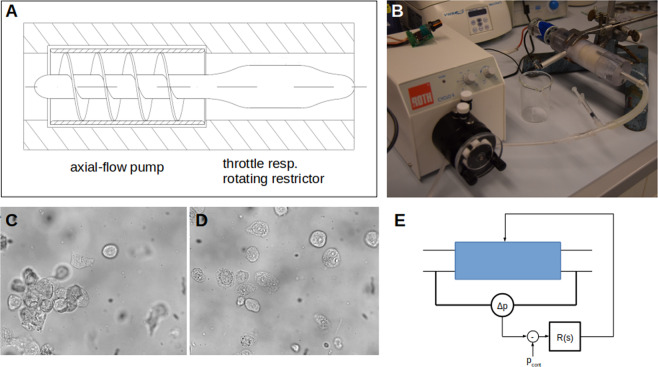
Design of a device to destroy CTCs. (**A**) General design. An axial-flow pump drives the blood containing cancer cells over the coaxial throttle which is a rotating restrictor. The two parts are designed in a way that no pressure difference between inlet and outlet occurs, i.e. the pressure built up by the axial-flow pump is completely dissipated by the throttle. Such a device was built (**B**) and cancer cell-clusters were processed through this device. As expected, clusters (**C**) were disintegrated (**D**). Also the viability is reduced by 93%. The schematic of the control circuit is shown in (**E**). The pressure difference (Δp) between inlet and outlet is measured and a control circuit (R(s)) regulates the motor speed in a way to bring Δp to a set value, which is normally set to 0.

Ultimately, to take a step further towards a setup that would be closer to a prototype of an implantable system, we designed a mechatronic device based on the very same principles but that pumps the blood with an axial pump over a coupled rotating restrictor (throttle) (Fig. 5A,B, Supplementary Figure 1 and Supplementary Animation 1). A roller pump, which simulates the heart, pumps medium containing cancer cell clusters through a nozzle, which corresponds to the resistance of the organs and subsequent vascular system, with the cancer cell-disruption device located in between (Fig. 5B). Before entering the device, cancer cells appear as clusters (Fig. 5C). Upon processing with 15 Pa for approximately 0.24 s however, virtually all clusters were destroyed (Fig. 5D) and the total number of viable cells was reduced by 93 ± 4% in 5 independent measurements. Also in this case, the rotational speed is controlled in a way that the pressure difference over the device is kept at zero (Fig. 5E), thus the circulatory system would not be influenced and the blood flow would also not be impaired. Finally, we measured the performance of this device, that allowed to reach higher rotational speed, using a shear stress of 25 Pa for 0.3 s which was achieved at a flow rate of 93 ml/min. In three independent measurements we destroyed all cancer cells in the sample. This holds true also for other cell lines like the BT474-cells (data not shown, ongoing study).

## Discussion

In the current report we show that both cancer cell clusters and single cancer cells are highly sensitive to fluid shear stress in comparison to blood cells. These findings suggest that fluid shear stress, when applied in a way that does not influence physiological circulation, could be an effective way to remove CTCs from a patient’s vascular system. CTC removal might be particularly relevant not only in the early setting (i.e. localized disease), but also in a more advanced setting whereby metastases have been shown to seed other metastases^21–23^. Additionally, we speculate that CTC removal might be beneficial also during primary surgery, since tumor manipulations may also trigger CTC release^24–31^.

Disruption of CTC-clusters as well as CTCs would be beneficial in two distinct cases:

(i) for a limited time after surgery (e.g. two weeks), when the risk of CTC-occurrence is highest and

(ii) permanently if an inoperable tumor producing CTCs is found in the patient.

With the purpose of proposing the concept of an implantable CTC-disrupting system, we designed a mechatronic device that pumps blood by an axial pump over a coupled rotating restrictor (throttle) and validated its activity using human breast cancer cells. This model allowed us to gain insights into the cancer cell-disrupting ability of our system. We found that the frequency (concentration) of cancer cells, which varies remarkable for different tumors^32^, has no influence onto the efficiency of cancer cell-cluster disintegration or cancer cell damage. However, deep knowledge of the flow rates and other haemodynamic parameters in various parts of the circulatory system will be required for a proper positioning and dimensioning of the device.

We are aware that additional models (including large animal models) that more closely resemble human CTCs in their physiological environment will be needed in future studies.

We found that two parameters are important to exert the appropriate shear stress onto cancer cells: A) The shear stress level and B) the shear stress duration. To account for different physiological blood flow in a patient, the rotational speed has to be controlled to keep the pressure difference over the device constant to 0. If lower flow is required, the revolution speed is decreased. This leads to a reduced shear stress at the throttle but on the other hand to increased duration of shear exposure due to the low flow. Conversely, high flow leads to shorter duration of stress exposure and concurrent increase in shear stress. In detail, around the working point of an axial pump, the volumetric flow *Q* (in m³/s) is in linear proportion to the rotational frequency *n, i.e*.

1 $$Q={K}_{1}\cdot n$$

With the empiric constant *K*_1_ summarizing the characteristics of the device.

The duration time *t*_*shear*_ in the gap of the throttle, i.e. the exposure time to shear stress is inverse proportional to the volumetric flow. Thus

2 $${t}_{{shear}}=\frac{A\cdot l}{Q}=\frac{A\cdot l}{{K}_{1}\cdot n}$$

with *A* being the cross section of the clearance and *l* the length of the throttle. On the other hand, the shear stress *σ*_*shear*_ is proportional to the rotational frequency yielding

3 $${\sigma }_{{shear}}=\eta \cdot \frac{U}{h}\cdot n$$

with *η* denoting the dynamic viscosity of the fluid, *U* being the circumference and h the height of the clearance of the throttle. The performance of CTC-disintegration (shear performance *Π*_shear_) was assumed to be

4 $${\varPi }_{{shear}}\approx {t}_{{shear}}\cdot {\sigma }_{{shear}}=\frac{A\cdot l}{{K}_{1}\cdot n}\cdot \eta \cdot \frac{U}{h}\cdot n=\eta \cdot \frac{U\cdot A\cdot l}{h\cdot {K}_{1}}$$

which resembles the simplest version of a fluid stress-based model for hemolysis^44^. Note that within a reasonable range, this shear performance is independent of the rotational speed n. Thus, for a given working point of the device, the desired volumetric flow or the local blood pressure respectively can be controlled by adjusting *n* while keeping the shear performance *Π*_shear_ constant.

**Figure 6:**
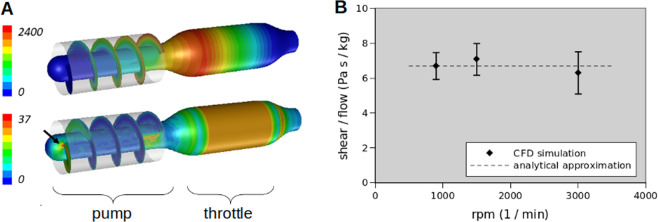
Evaluation of flow, pressure distribution, shear load and shear performance Π_shear_ by means of Computational Fluid Dynamic (CFD) simulations for blood. (**A**) The top contour plot of static pressure (all values given in Pa) indicates pressure increase in the rotating screw (1500 rpm) and subsequent pressure loss along the throttle. The bottom contour plot depicts wall-shear, indicating a local shear maximum at the leading edge of the screw (arrow) and a very homogeneous shear distribution in the throttle. (**B**) Ratio of shear and flow, averaged over the throttle length, evaluated for three operating conditions of 900, 1500 and 3000 rpm rotational speed, respectively. This ratio is proportional to the shear performance Π_shear_. The theoretical behavior according to Eq. 4 is shown as broken line indicating that indeed ovre a wide range of rotational speeds the shear performance is approximately constant. Blood has been considered as shear-thinning Carreau fluid^44^. CFD simulation have been performed on step-wise refined grids to rule out grid-dependencies. The pump-charaterizing constant K_1_ was obtained from linear fitting the flow-rotational speed dependence.

To further evaluate this behavior, which would be beneficial for practical application Computational Fluid Dynamics (CFD) simulations were carried out. This simulation allows for the consideration of all known effects, including the complex geometry as well as the non Newtonian behavior of blood. However, with one CFD-simulation only the (exact) behaviour of one particular parameter setting is calculated. Thus we use this method to verify, that the above found analytical behavior approximates the reality sufficiently well. A typical result is shown in Fig. 6. The pressure and shear stress distribution over the geometry is shown in Fig. 6A. Clearly the pressure is increased over the pump and then drops over the throttle so that the pressure difference over the whole device is 0. A very homogeneous shear stress distribution on the throttle can be seen, which is important for the correct function of the device in order to shear all the volume equally. In Fig. 6B the ratio of average shear stress throughout the throttle and the volumetric flow is plotted. This is proportional to the shear performance *Π*_shear_. As can be seen, this ratio is approximately constant for the rotational speeds tested, as could be expected from the analytical approximation given in Eq. 4.

At present, we generated a proof of concept device for a single pass of cancer cells or blood cells. We certainly envisage further improvements to allow the implantation of such a device in animal models, of course in a miniaturized form. Alternatively, this device could be operated extracorporeal, similar to haemodialysis, for a limited time after tumor surgery.

However, several restrictions have to be considered. For instance, the clearance of the throttle and the pump have to be designed in a way to avoid large turbulence and cavitation. The length of the throttle *l* determined the duration of shear application as derived above. Thus, it must be long enough to yield sufficient shear performance Π_shear_. Additionally, when planning the implantation of the device, all components must be made of haemocompatible materials and tested for a longer period of time to ensure that no blood cells are damaged and prolonged cancer cell-disruption is granted. In terms of coagulation and haemolysis, it might be assumed that our device behaves similar to axial pumps used for left ventricular assistive devices (LVAD). While typically in an artery the shear stress level is up to 10Pa^17,18^, these LVAD yield shear stress levels of 50 to 500Pa^33–36^. In these devices, only justifiable levels of haemolysis of 2–15% occur^37–43^. Detailed analyses of blood pumps were undertaken in the last years. For a great overview see for example Faghih *et al*.^44^. Furthermore these LVADs need to pump all the heart minute volume permanently through the body, whereas for cancer cell-disruption it might be sufficient to treat the blood in a vein downstream of an inflicted organ. This would affect only a fraction of the blood volume and thus yield even lower haemolysis. Concerning clotting it has to be mentioned that shear stress of about 25 Pa is shown to hardly or even not significantly induce clotting^45^ and 125 Pa induce clotting but in a way that can be handled using vitamin K-antagonists.

As we had the idea to use the device in the veins after the organ with the primary tumor, only a fraction of the heart-minute-volume will be pumped over the device. Thus the haemodynamic challenge as well as the technical problems should be further reduced in comparison to artificial hearts.

Taken together, our results provide a novel proof-of-concept device that enables the mechanical disruption of cancer cells and preservation of blood cells. To extend its validity, this approach will need to be carefully tested with other cancer cell types and in more complex experimental setups (e.g. implantation in living animals). Future experimental approaches should also be optimized to dissect the clinical utility of a CTC-disruption device, i.e. at which stage of tumor development the application of this technology can be beneficial. As shown by Hosseini *et al*.^46^, metastases may be derived from early disseminated cancer cells, thus timing of CTC disruption should be carefully evaluated. Nevertheless, our device demonstrates a high potential in metastasis prevention and upon optimization, its use might benefit patients who suffer from metastatic cancer.

## Materials And Methods

### Shear stress device

For the first measurements the setup (Fig. 1) consisted of independently controllable pump and throttle. The pump was a roller pump (Roth Cyclo II, Roth, Karlsruhe, Germany). The in-house built throttle was a cylinder made of Polyoxymethylen (POM) with a diameter of 10 mm and a length of 10 mm (excluding bearing and connection to the motor) in a POM-housing with a clearance of 0.1 mm. This cylinder was coupled to a DC-motor (ITEM#901623, Dickie-Tamiya Ltd., Fürth, Germany) with a custom RPM-control.

For the proof-of-concept we designed a device (Fig. 5) that rigidly couples an axial pump to the throttle. An axial impella-screw with attached casing pipe was 3D-printed using Acrylnitril-Butadien-Styrol-copolymers. The outer diameter of the casing pipe was 20 mm, the inner diameter of the pump-shaft was 10 mm. The length was 40 mm and we had a screw thread with two convolutions each performing 2 turns. The casing pipe was attached as we recently found in another project, where we tried to improve axial pumps for heart assisting devices, that the shear stress distribution is more uniform in such screw pumps than in open impella-pumps. The pump head (screw) was attached onto a shaft that was also connected to a subsequent throttle with 30 mm length and a clearance of 0.15 mm. Inflow and outflow sections were designed conically in order to avoid turbulence.

### Viability test

1 ml of cell suspension containing approximately 10^6^ cells/ml was injected for each condition into the device and then the device was washed with 1 ml of Hank’s Balanced Salt Solution (HBSS). The volume of 2 ml was collected and 20 µl of 5 mg/ml stock solution of neutral red were added. This suspension was incubated in an eppendorf-tube for 2 h at 37° with 5% CO_2_ and 100% humidity in an incubator. Then the suspension was centrifuged for 3 min at 210 g. The supernatant was disposed and the cells were washed with 1 ml HBSS. This was again centrifuged for 3 min at 210 g and the supernatant was again discarded. The cells were then suspended in 1 ml acidic ethanol (1% V/V glacial acetic acid in EtOH) and incubated for 15 min under slow overhead rotation. The suspension was centrifuged 3 min at 2100 g and the supernatant was photometrically measured at 540 nm (UV-1600PC spectral photometer, VWR, Vienna Austria). The background levels were detected by using non sheared cells which were killed initially using 0.1% TritonX-100 or alternatively 20 µg/ml KCN in the medium before being treated as the cells under investigation. For all conditions shown the viability tests were repeated 3 times using different cell batches.

Other methods for viability quantification were tested. However, as most cells were completely damaged by the device used and we can only see cell-debris, any microscopic or flow cytometry assay that requires “normally”-shaped cells fails. Thus the robust metabolic quantification as described above was used.

### Cell culture

MDA-MB-231 LM2 human breast cancer cells (obtained from Dr. Joan Massagué, MSKCC, NY, USA) were grown in Dulbecco’s Modified Eagle Medium (DMEM) (#11330-057, Gibco) supplemented with 10% Fetal Bovine Serum (FBS) (#10500064, Gibco) and antibiotic/antimycotic (#15240062, Gibco) in a humidified incubator at 37 °C with 20% O2 and 5% CO2. Cells were transduced with lentivirus carrying GFP-Luciferase (SBI, BLIV201PA-1-SBI). To obtain cell clusters, cells were trypsinized (#25200-056, Gibco) for max. 3 min and suspended in growth medium.

### Ethics statement and human blood test

Healthy donor blood was purchased from the Blutspendezentrum SRK beider Basel upon written informed consent of healthy volunteers. Healthy donor blood was divided into 4 samples to test 3 different shear conditions, where one sample served as a control. After collection from the device, blood was analyzed with an Advia120 Hematology Analyzer (Siemens) using Multispecies version 5.9.0-MS software (Bayer) to assess if the used shear stress conditions had an impact on the blood components.

### Mouse experiments

Mouse experiments were carried out according to institutional and cantonal guidelines (approved mouse protocol #2781, cantonal veterinary office of Basel-City). Nod Scid Gamma (NSG) mice were purchased from The Jackson Laboratory (Bar Harbor, Maine, USA) and kept in pathogen-free conditions, according to institutional guidelines.

1 ml of CTCs suspension containing 1×10^7^ cells was processed with the device for each tested condition. Then, collected fractions (100 µl, corresponding to 0.5×10^6^ cells per mouse) were injected in the tail vein of 6–8 weeks old mice and monitored weekly with IVIS Lumina II (Perkin Elmer). Metastatic burden was determined as a photon/second count. Lung dissection was performed after 5 weeks.

### Immunofluorescence

Frozen sections embedded in optimal cutting temperature compound (OCT) were cut as 10 μm sections and handled according to a standard immunofluorescent tissue staining protocol. Briefly, after 15 min fixing in 4% formaldehyde (freshly prepared from paraformaldehyde) and 1 hour of blocking, sections were stained using anti-GFP antibody (#2956, Cell Signaling) followed by incubation with secondary donkey anti-rabbit AF488 (#A-21206, Invitrogen) including DAPI (4′,6-diamidino-2-phenylindole) staining. Pictures were taken with the Leica DMI 6000 microscope.

## Supplementary information

Supplementary Figures.

Supplementary Animation.

## Data Availability

The datasets generated and/or analyzed during the current study are available from the corresponding author on reasonable request.
