# Supplementary material for: A novel device for elimination of cancer cells from blood specimens
Source: Sci Rep. 2020 Jun 23;10:10181. doi: 10.1038/s41598-020-67071-w (PMC7311454; doi:10.1038/s41598-020-67071-w)
Supplement: Supplementary file 1 — Supplementary Figures. [file 41598_2020_67071_MOESM1_ESM.pdf]

Supplementary information for:

A novel device for selective elimination of circulating tumor cells from blood specimens

Agnes Weth[1], Ilona Krol[2], Kurt Priesner[3], Cinzia Donato[2], Stefan Pirker[4], Christoph Wolf[1], Nicola Aceto[2*], Werner Baumgartner[1*]

[1] Institute of Biomedical Mechatronics, Johannes Kepler University of Linz, Altenbergerstr. 69, 4040 Linz, Austria
[2] Cancer Metastasis Laboratory, Department of Biomedicine, University of Basel and University Hospital Basel, 4058 Basel, Switzerland
[3] Griesmühle Ltd, Griesmühlweg 14, 4111 Walding, Austria
[4] Department of Particulate Flow Modelling, Johannes Kepler University of Linz, Altenbergerstr. 69, 4040 Linz, Austria

* To whom correspondence should be addressed:
Prof. Werner Baumgartner (werner.baumgartner@jku.at)
Prof. Nicola Aceto (nicola.aceto@unibas.ch)

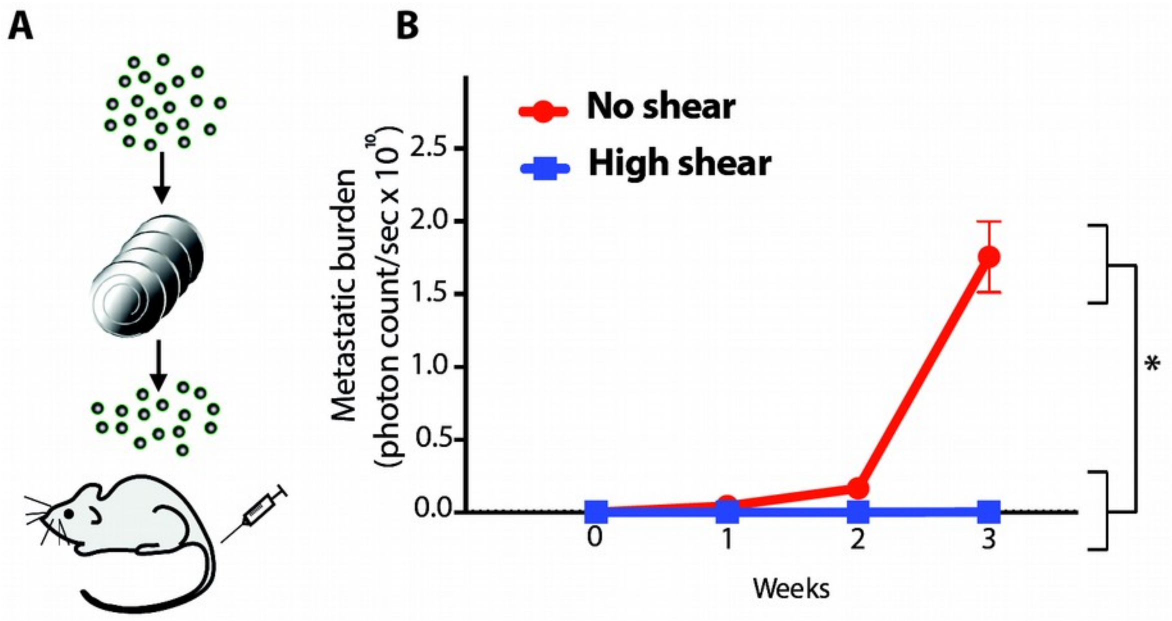

Supplementary Figure 1: Characterization of metastatic potential of sheared versus unsheared single cells. Schematic of experimental design. Single cells were exposed to shear stress, collected and injected in the tail vein of immunodeficient mice (A). The graph shows metastatic burden as a mean photon count/sec. n=4 for no shear, n=4 for high shear; error bars represent S.E.M., *P< 0.0004 by Student’s t test. (B).

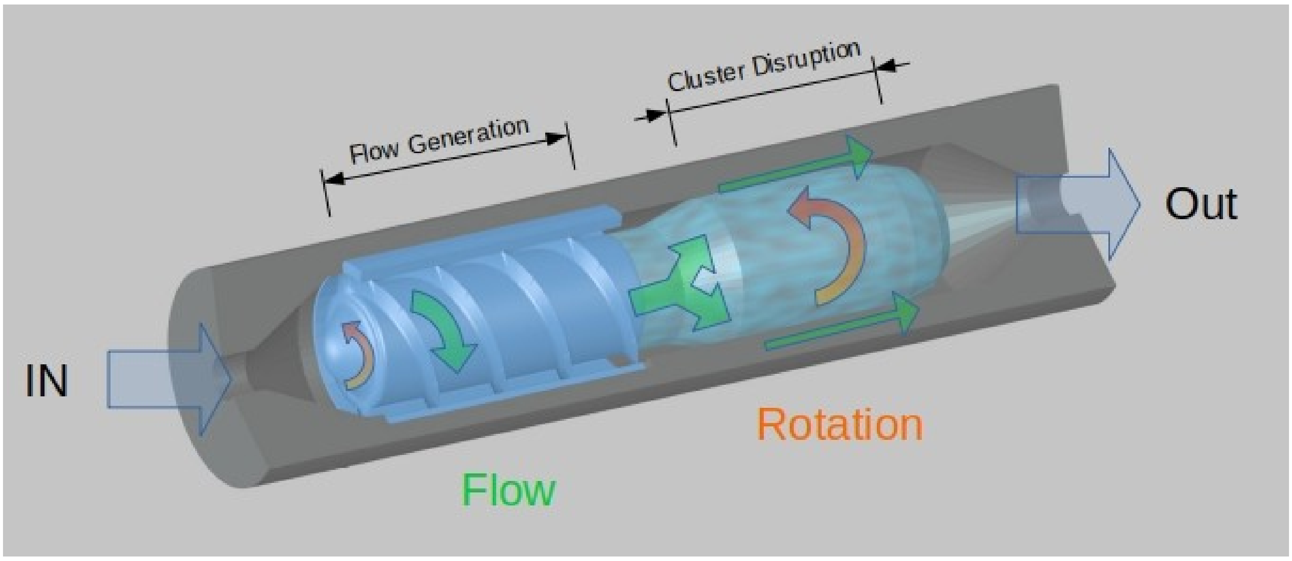

Supplementary Figure 2: Principal composition of our CTC-cluster disruption device. The axial pump (left) compensates the loss of flow rate caused by the rotating restrictor (right). Both parts are firmly connected and therefore the rotation speeds and directions are coupled. The restrictor serves as the actual cluster disruptor, by applying shear stress in two ways: It restricts the cross-sectional area available for blood-flow and, by means of its rotation, adds a momentum perpendicular to the flow direction.
